# Clinical and Demographic Profile of Patients Receiving Fingolimod in Clinical Practice in Germany and the Benefit–Risk Profile of Fingolimod After 1 Year of Treatment: Initial Results From the Observational, Noninterventional Study PANGAEA

**DOI:** 10.1007/s13311-017-0595-y

**Published:** 2017-12-22

**Authors:** Tjalf Ziemssen, Michael Lang, Björn Tackenberg, Stephan Schmidt, Holger Albrecht, Luisa Klotz, Judith Haas, Christoph Lassek, Jennie Medin, Christian Cornelissen, on behalf of the PANGAEA study group

**Affiliations:** 1 Center of Clinical Neuroscience, Neurological University Clinic Carl Gustav Carus, University of Technology, Dresden, Germany; 2 NeuroPoint Patient Academy and Neurological Practice, Ulm, Germany; 3 Department of Neurology, Clinical Neuroimmunology Group, Philipps-University, Marburg, Germany; 4 Bonn Neurological Practice, Bonn, Germany; 5 Neurological Practice, Munich, Germany; 6 Department of Neurology, University Hospital Münster, Münster, Germany; 7 Center for Multiple Sclerosis, Jewish Hospital Berlin, Berlin, Germany; 8 Kassel and Vellmar Neurological Practice, Vellmar, Germany; 9 Novartis Pharma AG, Basel, Switzerland; 10 Novartis Pharma GmbH, Nuremberg, Germany

**Keywords:** Multiple sclerosis, Fingolimod, Real-world evidence, Benefit–risk profile, Observational study

## Abstract

The population with multiple sclerosis receiving treatment in clinical practice differs from that in randomized controlled trials (RCTs). An assessment of the real-world benefit–risk profile of therapies is needed. This analysis used data from the large, noninterventional, observational German study Post-Authorization Non-interventional German sAfety study of GilEnyA (PANGAEA) to assess prospectively baseline characteristics and outcomes after 12 months (± 90 days) of fingolimod treatment. Patients were divided into 2 cohorts: fingolimod starter [first received fingolimod in PANGAEA (*n* = 3315)] and previous study [received fingolimod before enrollment in PANGAEA in RCTs (*n* = 875), some of whom also had baseline data at entry into RCTs (*n* = 505)]. At PANGAEA baseline, patients in the fingolimod starter *versus* the previous study cohort had a higher annualized relapse rate [ARR (95% confidence interval): 1.79 (1.75–1.83) *vs* 1.32 (1.25–1.40)] and Expanded Disability Status Scale score [3.11 (3.04–3.17) *vs* 2.55 (2.44–2.66)]. A greater proportion in the fingolimod starter *versus* previous study cohort had diabetes (2.0% *vs* 0.7%). After 12 months of fingolimod, ARRs were lower than in the 12 months before PANGAEA enrollment in the fingolimod starter [0.386 (0.360–0.414)] and previous study [0.276 (0.238–0.320)] cohorts. Expanded Disability Status Scale scores were stable *versus* baseline. Adverse events were experienced by similar proportions in both cohorts during fingolimod treatment. Relevant differences exist in disease activity and comorbidities between patients receiving fingolimod in clinical practice *versus* RCTs. Irrespective of baseline differences indicating a higher proportion at an advanced stage of multiple sclerosis in the real world *versus* RCTs, fingolimod remains effective, with a manageable safety profile.

**Electronic supplementary material:**

The online version of this article (10.1007/s13311-017-0595-y) contains supplementary material, which is available to authorized users.

## Introduction

Randomized controlled trials (RCTs) of disease-modifying therapies (DMTs) in multiple sclerosis (MS) are designed to evaluate treatment efficacy. In order to minimize the influence of confounding factors, such as concomitant diseases, RCTs often include a highly selected population of patients who are treated in specialist environments under optimal, restricted conditions [1–3]. RCTs generate high-quality data required for regulatory approval, but the experimental conditions under which they are conducted and the specific population that they often investigate mean that results may not be generalizable to the clinical use of DMTs in the real world [1, 4]. Following marketing authorization, robust real-world studies are needed to assess the safety and effectiveness of DMTs in the population of patients being treated in clinical practice, including those with characterized comorbidities and using concomitant medications [4–6].

In light of the need for robust real-world data following RCTs, the large, prospective, observational 5-year real-world Post-Authorization Non-interventional German sAfety study of GilEnyA (PANGAEA) has been initiated to investigate the effectiveness and safety of fingolimod 0.5 mg (Gilenya; Novartis Pharma AG, Basel, Switzerland) in clinical practice [7]. Real-world data can also be collected retrospectively from existing data sources, such as MS registries, including the international patient registry MSBase. However, such registries tend to focus on treatment effectiveness, and provide limited safety information, whereas PANGAEA has been designed to collect robust data for both safety and effectiveness [1, 7–9].

In the European Union (EU), fingolimod is indicated for patients with highly active MS, despite previous treatment with at least 1 DMT, and for individuals with rapidly evolving severe relapsing–remitting MS [10]. In contrast, the pivotal phase III RCTs included in the marketing authorization application to the European Medicines Agency for fingolimod [FTY720 Research Evaluating Effects of Daily Oral therapy in Multiple Sclerosis (FREEDOMS), FREEDOMS II, and Trial Assessing Injectable Interferon versus FTY720 Oral in Relapsing–Remitting Multiple Sclerosis (TRANSFORMS)] were designed to investigate fingolimod as a first-line DMT, which was the original intended label for fingolimod (see Table S1 for the eligibility criteria of these RCTs) [11–13]. Individuals in clinical practice who are eligible to receive fingolimod according to the EU label are therefore likely to have more advanced disease than those in the pivotal fingolimod RCTs, and PANGAEA will provide data with which to assess fingolimod in this real-world population [11–13].

Patients aged over 55 years with comorbidities such as diabetes mellitus and specified cardiovascular, pulmonary, hepatic, or autoimmune conditions, and those receiving certain concomitant medications were excluded from the 3 pivotal RCTs [11–13]. For PANGAEA, the only exclusion criteria were the contraindications listed in the European fingolimod Summary of Product Characteristics [10]. PANGAEA will therefore provide data for assessing the long-term benefit–risk profile of fingolimod in subgroups of patients with comorbidities or receiving concomitant medications who can receive the drug according to the EU label but who would have been excluded from clinical trials [7].

PANGAEA investigates all patients receiving fingolimod in clinical practice according to the EU label, including those who received the drug previously in RCTs and those starting therapy with it for the first time in clinical practice. PANGAEA provides a unique opportunity to compare directly the real-life safety and effectiveness of fingolimod in patients selected according to RCT criteria with that in individuals chosen using standard clinical practice criteria.

Herein, we describe the baseline characteristics of patients and the benefit–risk profile of fingolimod after 1 year of treatment in PANGAEA.

## Methods

### Study Design, Setting, and Patient Selection

PANGAEA is an ongoing, multicenter, prospective, noninterventional, observational long-term study [7]. All patients receiving fingolimod were eligible for inclusion provided they had a diagnosis of relapsing–remitting MS, had been prescribed fingolimod (0.5 mg daily) by their physician independently of study participation, and had provided informed written consent [7]. Recruitment took place at neurological practices and hospitals across Germany between April 2011 and December 2013, with the observational period expected to continue until December 2018 [7, 14]. There were no exclusion criteria, except the contradictions in the European fingolimod Summary of Product Characteristics [10].

An ethics committee was consulted prior to the study initiation and had jurisdiction over the medical director of the study. The study was conducted in accordance with the Declaration of Helsinki. Written informed consent was obtained from all participants in order to document his or her data prior to inclusion in the study.

### Patient Population and Study Cohorts

For this analysis, patients eligible for inclusion in PANGAEA who had 12 months (± 90 days) of follow-up were divided into cohorts based on their fingolimod experience at study entry. Patients receiving fingolimod for the first time were known as the “fingolimod starter cohort”. Those who had received fingolimod in clinical trials before PANGAEA were known as the “previous study cohort”. Some individuals in the previous study cohort had baseline data available from enrollment into previous fingolimod clinical trials, as well as at enrollment into PANGAEA; these patients were known as the “previous study subcohort”. The inclusion and exclusion criteria for clinical trials into which patients in the previous study cohort had been enrolled are shown in Table S1.

### Baseline Characteristics and Study Outcomes

At PANGAEA enrollment, comorbidities of interest [defined as comorbidities of potential interest to neurologists based on the adverse events (AEs) that patients may be at increased risk of experiencing during treatment with fingolimod, as well as on contraindications for fingolimod, as outlined in the fingolimod Summary of Product Characteristics [10]], concomitant medications, and demographic and clinical characteristics [Expanded Disability Status Scale (EDSS) score, annualized relapse rate (ARR) in the 12-month prebaseline period, and disease severity (number of T2 or gadolinium-enhancing magnetic resonance imaging lesions)] were recorded by the treating neurologist during interviews or medical examinations. According to routine practice, and as recommended by the German Society of Neurology and the fingolimod Summary of Product Characteristics, patient visits were scheduled at baseline, at month 1, and every 3 months thereafter [7, 10].

Clinical effectiveness was evaluated after 12 months (± 90 days) of fingolimod treatment in all patients included in the analysis. Clinical effectiveness data were collected by the treating neurologist at each visit. Outcomes investigated included change in disability from baseline in EDSS score, ARR, and the proportion of patients free from relapses and with 6-month confirmed disability worsening. Patients were classified as having experienced a relapse by the treating physician according to their clinical judgment at each patient visit. There were no predefined criteria for a patient to be classified as having experienced a relapse in this study. Relapses were documented at the time of patient visits, beginning at month 1 [7]. Confirmed disability worsening was determined according to increases in EDSS score from baseline, with confirmation of the increase in disability at a visit in the absence of a relapse. For 6-month confirmed disability worsening, the initial EDSS score at onset of disability progression, the 6-month confirming EDSS score, and all EDSS evaluations in between needed to have met the disability progression criteria.

AEs and serious AEs (SAEs) were evaluated after 12 months (± 90 days) of fingolimod treatment in all patients included in the analysis. An AE was defined as any unfavorable change in a patient’s pretreatment condition, regardless of a potential relationship to treatment and irrespective of whether medication was taken as intended. SAEs were defined as lethal or life-threatening events, hospitalizations, events leading to major incapacity, persistent or significant disability or incapacity, congenital anomaly or birth defect, and events that were otherwise deemed to be medically significant (e.g., abnormal laboratory values or test results). At every visit, the treating neurologist evaluated and documented the occurrence of AEs and SAEs. For each event, the type, time of first occurrence, duration, intensity, and causal relationship to the therapy were documented. AEs and SAEs were classified using the Medical Dictionary for Regulatory Activities [7].

### Statistical Analysis

For categorical variables, data are presented as the number of cases and the proportion of cases in each category. For continuous variables, data are summarized using the mean, 95% confidence interval (CI), SD, and median. For proportions of patients, 95% CIs were calculated using the exact (Clopper–Pearson) method. For relapses, ARRs and associated 95% CIs were analyzed using a negative binomial distribution model and the logarithm of the time on study as an offset variable. Relapses were not included in this analysis if they occurred within 30 days of a previous relapse that had already been included. Patients for whom MS was a cause of death were considered to have confirmed disability worsening, regardless of the baseline EDSS score or the change in EDSS score. For comorbidities, concomitant medications, AEs, and SAEs, if patient data were missing, or if patients were lost to follow-up, data were taken into consideration up to the point of discontinuation; for ARR and EDSS analysis, patients were excluded from the analysis if they did not have data available after 12 months (± 90 days) of fingolimod treatment.

## Results

### Study Population

**Fig. 1:**
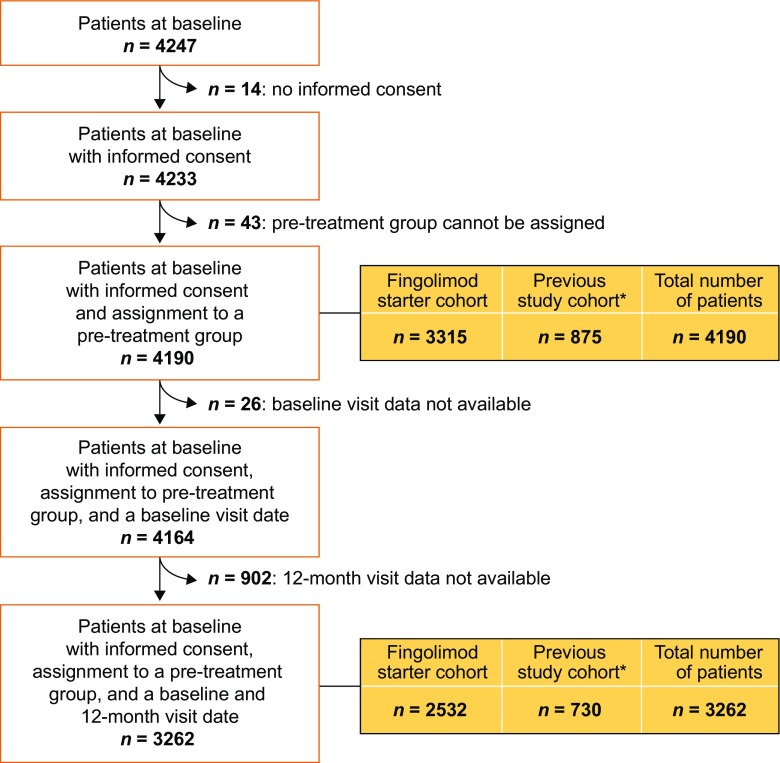
Patient flow diagram. *Patients in the previous study subcohort (*n* = 505) were identified in randomized controlled trials according to patient number, sex, and year of birth between Post-Authorization Non-interventional German sAfety study of GilEnyA (PANGAEA) and previous fingolimod clinical trials. Patients with no match according to these criteria were excluded from the previous study subcohort

Patients were recruited into PANGAEA from 374 neurological centers across Germany (Fig. S1). A total of 4190 patients met the criteria for inclusion in PANGAEA; of these, 3315 comprised the fingolimod starter cohort and 875 comprised the previous study cohort (Fig. 1). Of patients included in the previous study cohort, 505 also had baseline data available at entry into previous fingolimod clinical trials (previous study subcohort). In the previous study cohort, the mean ± SD treatment gap between the end of the RCT and enrollment in PANGAEA was 28.3 ± 102.5 days [15].

The highest proportion of patients was recruited from office-based neurologists (66.3%), followed by universities (13.2%), hospitals (13.1%), and multidisciplinary clinics (7.4%) (Table S2). The observed dropout rate during the 12-month follow-up period was 13.1% (10.3% was a result of therapy discontinuation and 2.8% was a result of study discontinuation).

### Baseline Characteristics

Baseline demographic and clinical characteristics are shown in Table 1. The age distribution of patients (Fig. S2) and duration of MS at entry into PANGAEA were similar across cohorts. At PANGAEA baseline, patients in the fingolimod starter cohort had numerically higher ARRs in the 12-month period before study start than those in the previous study cohort and those in the previous study subcohort. Patients in the fingolimod starter cohort also had higher EDSS scores than those in the previous study cohort and previous study subcohort at PANGAEA baseline; the distribution of EDSS scores in each of the cohorts is shown in the appendix (Fig. S3). The number of gadolinium-enhancing lesions was also greater in patients in the fingolimod starter cohort than in the previous study cohort and previous study subcohort at PANGAEA baseline. For patients in the previous study subcohort, ARRs in the 12-month period before the start of RCTs, and EDSS scores at RCT baseline were similar to those at PANGAEA baseline for individuals in the previous study cohort but were numerically lower than for those in the fingolimod starter cohort.

**Table 1:** Baseline demographic and clinical characteristics for each patient cohort

	PANGAEA baseline			RCT baseline
	Fingolimod starter cohort (*n* = 3315)	Previous study cohort (*n* = 875)	Previous study subcohort (*n* = 505)*	Previous study subcohort (*n* = 505)*
Demographics				
Mean ± SD age (y)	39.2 ± 10.1	40.3 ± 9.5	40.0 ± 9.3	38.8 ± 9.3
Median	39.5	40.9	40.9	39.0
Female	2351 (70.9)	641 (73.3)	372 (73.7)	372 (73.7)
Mean ± SD duration of MS (y)^†^	8.4 ± 6.6	8.1 ± 6.1	7.8 ± 5.9	7.0 ± 5.8
Previous MS DMTs				
No previous DMT	176 (5.3)	79 (9.0)	44 (8.7)	36 (7.1)
IFNs	1578 (47.6)	433 (49.5)	280 (55.4)	300 (59.4)
Glatiramer acetate	779 (23.5)	183 (20.9)	90 (17.8)	156 (30.9)
Natalizumab	617 (18.6)	137 (15.7)	73 (14.5)	77 (15.2)
Clinical characteristics				
Mean ARR (95% CI)	1.79 (1.75–1.83)	1.32 (1.25–1.40)^‡^	1.18 (1.09–1.28)^‡^	1.20 (1.10–1.30)
Mean EDSS score (95% CI)	3.11 (3.04–3.17)	2.55 (2.44–2.66)	2.43 (2.29–2.58)	2.37 (2.24–2.50)
T2-weighted lesions				
No lesions	159 (4.8)	63 (7.2)	39 (7.7)	NA
1–9	361 (10.9)	89 (10.2)	56 (11.1)	NA
> 9	2617 (78.9)	656 (75.0)	366 (72.5)	NA
Missing data	178 (5.4)	67 (7.7)	44 (8.7)	NA
Gd-enhancing lesions				
No lesions	1879 (56.7)	597 (68.2)	350 (69.3)	NA
1–9	910 (27.5)	132 (15.1)	72 (14.3)	NA
> 9	321 (9.7)	73 (8.3)	37 (7.3)	NA
Missing data	205 (6.2)	73 (8.3)	46 (9.1)	NA

Data are *n* (%) unless otherwise indicated. PANGAEA = Post-Authorization Non-interventional German sAfety study of GilEnyA; RCT = randomized controlled trial; MS = multiple sclerosis; DMT = disease-modifying therapy; IFN = interferon; ARR = annualized relapse rate; CI = confidence interval; EDSS = Expanded Disability Status Scale; NA = not available; Gd = gadolinium

*The proportion of patients in the previous study cohort who had baseline data available at enrollment into previous fingolimod clinical trials and at enrollment into PANGAEA

^†^Duration of MS since diagnosis

^‡^Patients in the previous study cohort had to discontinue fingolimod treatment between the end of RCTs and enrollment in PANGAEA; the mean ± SD gap between treatments was 28.3 ± 102.5 days

“Depression and mood disorders” was the most commonly reported comorbidity of interest at PANGAEA baseline (fingolimod starter cohort: 9.5%; previous study cohort: 12.6%; previous study subcohort: 13.0% Table S3), followed by hypertension (7.4%, 11.0%, and 11.4%, respectively). The proportion of patients with diabetes at PANGAEA baseline was highest in the fingolimod starter cohort (2.0%) but similar in the previous study cohort (0.7%) and previous study subcohort (0.7%). For concomitant medications of interest, analgesics were used by the greatest proportion of patients at PANGAEA baseline (fingolimod starter cohort: 13.4%; previous study cohort: 13.2%; previous study subcohort: 13.5% Table S4). The proportion of patients using fampridine at PANGAEA baseline was highest in the fingolimod starter cohort (2.3%) followed by the previous study cohort (0.9%) and then the previous study subcohort (0.5%).

### Effectiveness Outcomes at 12 Months

**Fig. 2:**
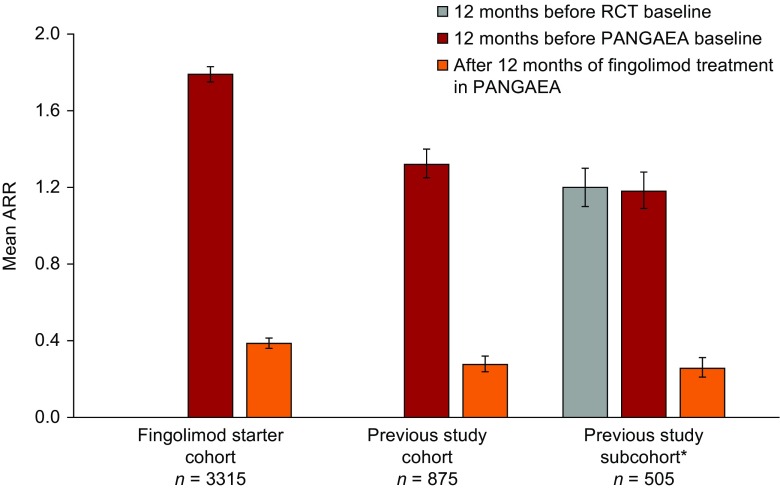
Annualized relapse rate (ARR) in the 12 months before study start and after 12 months of fingolimod treatment in PANGAEA. PANGAEA = Post-Authorization Non-interventional German sAfety study of GilEnyA; RCT = randomized controlled trial. *The proportion of patients in the previous study cohort who had baseline data available at enrollment into previous fingolimod clinical trials and at enrollment into PANGAEA. Error bars show 95% confidence intervals

After 12 months of fingolimod treatment, ARRs were lower in the fingolimod starter cohort [ARR: 0.386 (95% CI 0.360–0.414)] and the previous study cohort [ARR: 0.276 (95% CI 0.238–0.320)] than in the 12-month period before PANGAEA enrollment (Fig. 2). For patients in the previous study subcohort, ARRs after 12 months of fingolimod treatment [ARR: 0.256 (95% CI 0.210–0.312)] were numerically lower than those in the 12 months before PANGAEA or RCT enrollment.

**Fig. 3:**
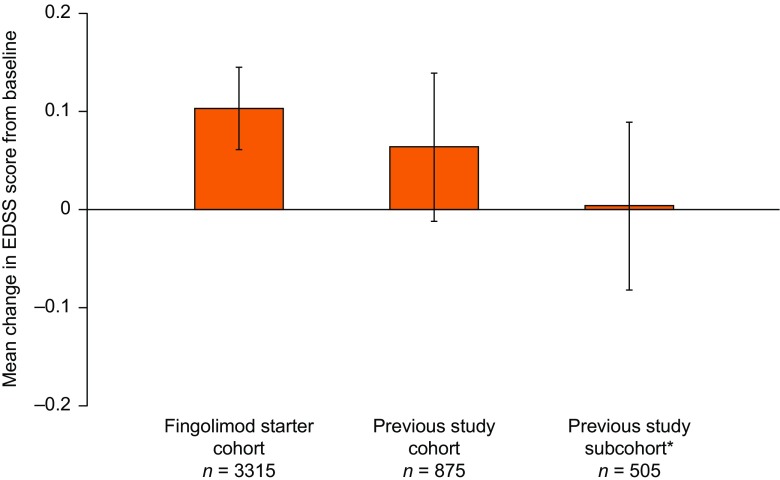
Change from baseline in Expanded Disability Status Scale (EDSS) scores after 12 months of fingolimod treatment in PANGAEA. PANGAEA = Post-Authorization Non-interventional German sAfety study of GilEnyA. *The proportion of patients in the previous study cohort who had baseline data available at enrollment into previous fingolimod clinical trials and at enrollment into PANGAEA. Error bars show 95% confidence intervals

**Fig. 4:**
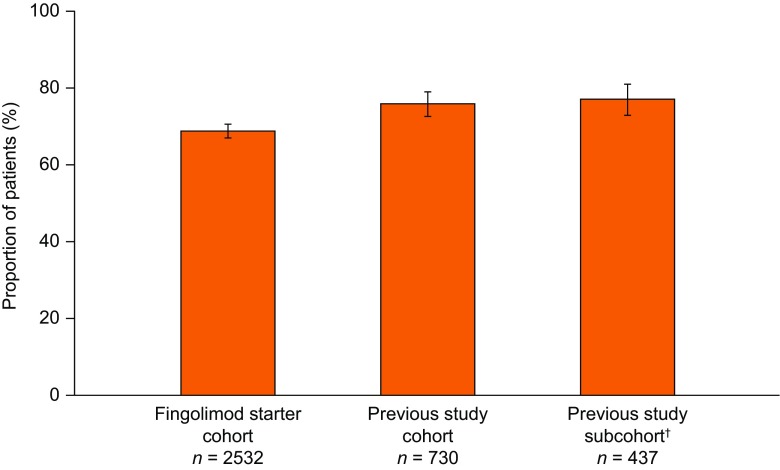
Proportion of patients free from relapses and 6-month confirmed disability worsening after 12 months of fingolimod treatment in PANGAEA*. PANGAEA = Post-Authorization Non-interventional German sAfety study of GilEnyA. *Confirmed disability worsening was assessed according to increases in Expanded Disability Status Scale (EDSS) score from baseline, with confirmation of the increase in disability made at a visit in the absence of a relapse: a 1.5-point increase from a baseline EDSS score of 0; a 1-point increase from baseline EDSS scores of 1–5.0; and a 0.5-point increase in baseline EDSS scores of 5.5 or more. ^†^The proportion of patients in the previous study cohort who had baseline data available at enrollment into previous fingolimod clinical trials and at enrollment into PANGAEA. Error bars show 95% confidence intervals

EDSS scores remained stable after 12 months of treatment compared with PANGAEA baseline in the fingolimod starter cohort [change in EDSS scores from baseline: +0.103 (95% CI +0.061 to +0.145)] and the previous study cohort [+0·064 (95% CI –0.012 to +0.139); Fig. 3]. For patients in the previous study subcohort, EDSS scores after 12 months of treatment were also stable compared with PANGAEA baseline [+0.004 (95% CI –0.082 to +0.089)]. EDSS scores were numerically lower in the previous study cohort and previous study subcohort than in the fingolimod starter cohort after 12 months of treatment. A higher proportion of patients were free from relapses and 6-month confirmed disability worsening following 12 months of fingolimod treatment in PANGAEA in the previous study cohort [75.9% (95% CI 72.6–79.0)] and previous study subcohort [77.1% (95% CI 72.9–81.0)] than the fingolimod starter cohort [68.8% (95% CI 67.0–70.6); Fig. 4].

### Safety Outcomes at 12 Months

During the first 12 months of fingolimod treatment, AEs were experienced by 21.5% of patients in the fingolimod starter cohort, 29.7% of those in the previous study cohort, and 30.9% of those in the previous study subcohort; for SAEs, the corresponding proportions were 3.8%, 4.2%, and 3.2%, respectively. For AEs of special interest (Table 2), hypertension was the most frequent event in the fingolimod starter cohort (1.6%) and the previous study cohort (2.2%). In the previous study subcohort, the most frequent event was increased alanine aminotransferase level (2.0%), followed by hypertension (1.3%).

**Table 2:** Adverse events (AEs) of special interest as a proportion of the total number of AEs in each cohort after 12 months of fingolimod treatment

System	Preferred term	Number of AEs (%)		
		Fingolimod starter cohort*	Previous study cohort*^,†^	Previous study subcohort*^,†^
Cardiac events	Hypertension	15 (1.6)	10 (2.2)	4 (1.3)
	Bradycardia	0 (0)	0 (0)	0 (0)
	Atrioventricular block, second degree	0 (0)	0 (0)	0 (0)
	Atrioventricular block, first degree	0 (0)	0 (0)	0 (0)
	Electrocardiogram QT prolonged	0 (0)	0 (0)	0 (0)
Infections	Herpes zoster	3 (0.3)	1 (0.2)	1 (0.3)
	Progressive multifocal leukoencephalopathy	0 (0)	0 (0)	0 (0)
	Meningitis, cryptococcal	0 (0)	0 (0)	0 (0)
Leukopenia	Lymphopenia	0 (0)	2 (0.4)	1 (0.3)
	Leukopenia	1 (0.1)	0 (0)	0 (0)
	WBC count decreased	0 (0)	0 (0)	0 (0)
Diseases of the nervous system	Posterior reversible encephalopathy syndrome	0 (0)	0 (0)	0 (0)
	Acute disseminated encephalomyelitis	0 (0)	0 (0)	0 (0)
Hepatic enzymes	Hepatic enzyme level increased	5 (0.5)	1 (0.2)	1 (0.3)
	ALT level increased	3 (0.3)	6 (1.3)	6 (2.0)
Eye disorder	Macular edema	0 (0)	0 (0)	0 (0)
Carcinoma	BCC	0 (0)	0 (0)	0 (0)
	Malignant melanoma	0 (0)	0 (0)	0 (0)
	Malignant melanoma *in situ*	0 (0)	0 (0)	0 (0)
	Neoplasm skin	0 (0)	0 (0)	0 (0)
	Penile SCC	0 (0)	0 (0)	0 (0)
	SCC of the vulva	0 (0)	0 (0)	0 (0)
Lymphoma	Diffuse large B-cell lymphoma stage I	0 (0)	0 (0)	0 (0)
	Follicle center lymphoma (follicular grade I, II, III stage IV)	0 (0)	0 (0)	0 (0)
	Non-Hodgkin lymphoma	0 (0)	0 (0)	0 (0)
Pregnancy	Abortion, spontaneous	0 (0)	0 (0)	0 (0)
	Abortion	0 (0)	0 (0)	0 (0)
	Abortion, early	0 (0)	0 (0)	0 (0)
	Abortion, incomplete	0 (0)	0 (0)	0 (0)
	Abortion, induced	1 (0.1)	0 (0)	0 (0)
	Ectopic pregnancy	0 (0)	0 (0)	0 (0)
	Exposure during pregnancy	0 (0)	0 (0)	0 (0)

WBC = white blood cells; ALT = alanine aminotransferase; BCC = basal cell carcinoma; SCC = squamous cell carcinoma.

*Not all patients experienced an AE in the first 12 months of receiving fingolimod in Post-Authorization Non-interventional German sAfety study of GilEnyA (PANGAEA). The total numbers of patients reporting an AE were as follows: fingolimod starter cohort, *n* = 2532; previous study cohort, *n* = 730; previous study cohort with baseline data available from previous clinical trials, *n* = 437

^†^The proportion of patients in the previous study cohort who had baseline data available at enrollment into previous fingolimod clinical trials and at enrollment into PANGAEA

## Discussion

Real-world studies provide insight into the postapproval benefit–risk profile of DMTs according to their clinical use in the population of patients eligible to receive treatment in routine practice. Such studies are an important follow-on from RCTs, which are often conducted under experimental conditions in a specific population of patients in order to limit the impact of confounding factors when investigating the efficacy of DMTs [6]. The need for real-world studies is illustrated by the present analysis, which used data from the German noninterventional, observational study PANGAEA to evaluate baseline characteristics and outcomes after 12 months of fingolimod therapy in patients receiving treatment and being monitored according to standard clinical practice. Importantly, PANGAEA differs from many other real-world studies in that it provides extensive data with which to evaluate comorbidities and concomitant medications used by patients, as well as the safety of fingolimod during treatment. Furthermore, this study is novel in that it allows for direct comparisons to be made between a cohort of patients receiving fingolimod in clinical practice who met the inclusion criteria of previous fingolimod RCTs (previous study cohort) and those receiving fingolimod for the first time in PANGAEA according to its EU label (fingolimod starter cohort).

In the present study, patients receiving treatment in RCTs and those being treated in clinical practice had similar ages and disease durations at PANGAEA enrollment. However, individuals in the fingolimod starter cohort had higher baseline disease activity than those in the previous study cohort (according to ARRs, EDSS scores, and the number of gadolinium-enhancing lesions), highlighting the potential for differences between patients in RCTs and those in clinical practice. A possible reason for these differences between cohorts is that a higher proportion of patients in the previous study cohort would have received fingolimod as a first-line DMT in RCTs, whereas individuals in the fingolimod starter cohort are more likely to have experienced disease activity on at least 1 previous DMT in accordance with the EU label [6]. The lower level of baseline disease activity in the previous study cohort compared with the fingolimod starter cohort may also be attributed to selection bias as patients who benefited from fingolimod in RCTs would be more likely to go on to receive it in PANGAEA.

The eligibility criteria of RCTs could potentially exclude patients with comorbidities and those receiving concomitant medications who may be eligible to receive fingolimod in clinical practice. At PANGAEA enrollment, a higher proportion of patients in the fingolimod starter cohort than the previous study cohort had diabetes and more patients in the fingolimod starter cohort than the previous study cohort were using fampridine and antidiabetics. These differences most likely occur because of the unrestrictive eligibility criteria of PANGAEA compared with those of fingolimod RCTs (Table S1). For example, patients with diabetes were excluded from the 3 pivotal fingolimod RCTs [11–13] owing to the potentially increased risk of macular edema [7], but were eligible to receive fingolimod in clinical practice and were therefore included in PANGAEA. Hence, PANGAEA offers the opportunity to reassess the real-world relevance of previously identified risk factors associated with fingolimod treatment, such as macular edema in patients with diabetes mellitus.

Patient baseline characteristics can potentially influence treatment outcomes [16]. Importantly, however, in the present study, irrespective of differences in disease severity, comorbidities, or concomitant medication use at baseline, ARRs were reduced after 12 months of fingolimod treatment compared with during the 12-month period before PANGAEA enrollment. In addition, EDSS scores remained stable, and a high proportion of patients were free from relapses and 6-month confirmed disability worsening across the cohorts. The effectiveness of fingolimod observed in the present study is therefore consistent with data from phase 3 RCTs, despite clear differences in patient’s baseline characteristics pointing towards more advanced disease at baseline in the fingolimod starter cohort than the previous study cohort [11–13].

The safety profile of fingolimod should be considered in the context of comorbidities that patients with MS may be at increased risk of developing and concomitant medications that are used frequently [17]. For example, in the present study, concomitant medications most frequently used by patients across the cohorts were analgesics and antidepressants. Open-label studies have demonstrated a relationship between the use of some drugs with cardiac effects (antidepressants, anticonvulsant/antimigraine, and antifatigue agents) and a need for extended first-dose monitoring [18–20]. In the present study, irrespective of differences in baseline characteristics, AEs of special interest occurring with the highest frequency across cohorts were hypertension and increased alanine aminotransferase level. These AEs are consistent with those reported in RCTs, with no new safety concerns being raised [11–13, 17], and are also consistent with findings in real-world studies demonstrating that patients with MS are at increased risk of certain metabolic and cardiovascular diseases [17, 21–23].

A strength of this study is that it presents data reflecting the clinical use of fingolimod as a second-line therapy for a large number of patients from different types of neurological centers across Germany, and it also allows for the safety profile of fingolimod to be evaluated extensively in clinical practice. A limitation of this study is that the data may not be generalizable owing to the fact that they reflect the baseline characteristics and use of fingolimod within the German population according to its EU label. It may therefore not be possible to extrapolate these findings to other countries, particularly those in which fingolimod use is restricted or the approved label indication is different. Nevertheless, the healthcare system in Germany does not restrict patient access to fingolimod, so it is fully reimbursed as indicated in the label. As a result, data generated in PANGAEA present the benefit–risk profile of fingolimod within its approved EU indication in a diverse population of patients.

Using PANGAEA as a case study, this report highlights the importance of real-world studies in complementing and expanding knowledge gained from the experimental setting of RCTs by demonstrating the benefit–risk profile of DMTs according to their use in clinical practice. Importantly, PANGAEA differs from many other real-world studies in that it collects data at baseline on comorbidities and concomitant medication use, and evaluates extensively the safety profile of fingolimod. This analysis demonstrates that there are differences in baseline characteristics between patients receiving fingolimod in clinical practice and in clinical trials. Irrespective of baseline differences, fingolimod was shown to be an effective therapy with a manageable safety profile. PANGAEA investigates a broad population of well-characterized patients receiving fingolimod in clinical practice, which will provide the opportunity to investigate subpopulations excluded from RCTs and to compare outcomes between cohorts with different characteristics (e.g., early-stage *vs* late-stage disease).

## Electronic supplementary material

ESM 1 (DOCX 47 kb)

ESM 2 (GIF 118 kb)

High resolution image (EPS 2603 kb)

ESM 3 (GIF 36 kb)

High resolution image (EPS 2148 kb)

ESM 4 (GIF 26 kb)

High resolution image (EPS 2128 kb)

ESM 5 (PDF 515 kb)
