# Supplementary material for: Clinical and Demographic Profile of Patients Receiving Fingolimod in Clinical Practice in Germany and the Benefit–Risk Profile of Fingolimod After 1 Year of Treatment: Initial Results From the Observational, Noninterventional Study PANGAEA
Source: Neurotherapeutics. 2017 Dec 22;15(1):190–9. doi: 10.1007/s13311-017-0595-y (PMC5794706; doi:10.1007/s13311-017-0595-y)
Supplement: Supplementary file 1 — (DOCX 47 kb) [file 13311_2017_595_MOESM1_ESM.docx]

Appendix: supplementary material

Table S1 Inclusion and exclusion criteria of previous randomized controlled trials into which patients in the previous study cohort had been enrolled*

| **Study name** | | **FREEDOMS** | **TRANSFORMS** | | **UMBRELLA** | | **FIRST** | | **TOFINGO** | **BIOBANK** | | **REGAIN** | | **STAY** |
| --- | --- | --- | --- | --- | --- | --- | --- | --- | --- | --- | --- | --- | --- | --- |
| ClinicalTrials·gov number | | NCT00289978 | NCT00340834 | | NCT01201356 | | NCT01127750 | | NCT01499667 | | NCT01310166 | NCT01436643 | | NCT01709812 |
| Inclusion criteria | | | | | | | | | | | | | | |
| MS | | RRMS | RRMS | | – | | | RMS | RRMS | | RMS | | RRMS | RRMS |
| Age, years | | 18–55 | 18–55 | | – | | | 18–65 | 18–65 | | 18–65 | | 18–65 | 18–65 |
| EDSS score | | 0–5.5 | 0–5.5 | | – | | | – | 0–6.0 | | 0–6.5 | | 0–6.5 | 0–6.5 |
| Disease activity | | No relapse  ≤ previous  30 days | No relapse  ≤ previous  30 days | | – | | | - |  | | – | | Defined by the fingolimod label | Defined by the fingolimod label |
| Pregnancy test | | Negative | Negative | | Negative | | | Negative | Negative | | Negative | | Negative | Negative |
| Contraception | | Active | Active | | Active | | | Active | Active | | Active | | Active | Active |
| Study-specific condition | | One relapse  ≤ previous 12 months or two relapses ≤ previous 24 months | One relapse  ≤ previous 12 months or two relapses ≤ previous 24 months | | Completion of a previous fingolimod study | | | – | Discontinued natalizumab | | – | | Mild to moderate depression | Patient treated with fingolimod for ≥ 6 months |
| Exclusion criteria | | | | | | | | | | | | | | |
| Chronic diseases of the immune system | x | | | x | | x | | X | – | | x | | X | Fingolimod label |
| Epileptic seizures^†^ | – | | | x | | – | | – | – | |  | | – | – |
| History of malignancies (except BCC or SCC) | x | | | x | | – | | ≤ the past 5 years | X | | ≤ the past 5 years | | X | ≤ the past 5 years |
| Diabetes mellitus | x | | | x | | Moderate/ severe or uncontrolled | | Moderate/ severe or uncontrolled | Uncontrolled | | Moderate/ severe or uncontrolled | | – | Fingolimod label |
| Active macular edema | – | | | x | | – | | X | X | | x | |  | Fingolimod label |
| History of retinal vascular diseases associated with macular edema | x | | | – | | – | | – | – | | – | | – | Fingolimod label |
| Active systemic infections^‡^ | x | | | x | | X | | X | x (Severe) | | x | | X | Fingolimod label |
| Total lymphoid irradiation or bone marrow transplant | x | | | x | | – | | X | X | | x | | X | Fingolimod label |
| Steroid treatment§ | x | | | x | | – | | X | X | | x | | X | Fingolimod label |
| Previous cladribine, cyclophosphamide, or mitoxantrone treatment | x | | | x | | X | | X | Cladribine or mitoxanthrone | | x | | Cladribine any time; cyclophosphamide, mitoxanthrone  ≤ 6 months | Fingolimod label |
| Previous immunosupressants | ≤ previous 6 months | | | ≤ previous  6 months | | – | | ≤ previous  3 months | ≤ previous  3 months | | ≤ previous 3 months | | ≤ previous 3 months | Fingolimod label |
| Treatment with IFNs or GA^†^ | x | | | – | | – | | – | – | | – | | – | Fingolimod label |
| Treatment with immunoglobulins or monoclonal antibodies^†^ | x | | | x | | X | | X | x (excluding natalizumab) | | x | | X | Fingolimod label |
| Certain defined cardiovascular condition | x | | | x | | X | | X | X | | x | | X | Fingolimod label |
| Certain defined pulmonary conditions | x | | | x | | X | | Pulmonary fibrosis; tuberculosis | X | | Pulmonary fibrosis; tuberculosis | | – | Fingolimod label |
| Certain defined hepatic conditions | x | | | x | | Alcohol abuse and chronic liver disease | | X | X | | x | | Severe hepatic dysfunction | Fingolimod label |
| Certain defined abnormal laboratory values | x | | | x | | – | | X | X | | x | | X | Fingolimod label |
| Certain neurological/ psychiatric disorders | x | | | x | | – | | X | – | | x | | X | Fingolimod label |
| Unable to undergo MRI scans | x | | | x | | – | | – | X | | – | | – | – |
| Pregnant or nursing | x | | | x | | X | | X | X | | x | | X | x |
| Previous treatment with fingolimod | x | | | x | | – | | X | X | | x | | X | – |
| Participation in another study^‖^ | x | | | x | | – | | X | – | | x | | X | x |
| Any medically unstable condition^¶^ | x | | | x | | X | | X | X | | x | | X | x |
| Negative VZV test | – | | | – | | – | | X | X | | x | | X | Fingolimod label |
| Live or live attenuated vaccines^**^ | – | | | – | | – | | X | – | | x | | X | Fingolimod label |
| Crohn’s disease or ulcerative colitis | – | | | – | | – | | – | X | | – | | X | Fingolimod label |
| Antidepressants^§^ | – | | | – | | – | | – | – | | – | | X | – |

^*^Further information about the study protocols is available from Clinicaltrials.gov. ^†^≤ previous 3 months. ^‡^Active systemic bacterial, viral, or fungal infections (including HIV and hepatitis B and C). ^§^≤ previous 30 days. ^‖^With any investigational drug ≤ previous 6 months. ^¶^That may interfere with the patient’s ability to cooperate and comply with the study procedures, as assessed by the treating physician. ^**^Including for VZV or measles ≤ previous 2 months. – = not listed in the inclusion or exclusion criteria; BCC = basal cell carcinoma; BIOBANK = Biomarker Study After Initiation of Treatment With Fingolimod (FTY720) in Patients With RRMS; EDSS = Expanded Disability Status Scale; FIRST = Tolerability and Safety and Health Outcomes in RMS Patients; FREEDOMS = FTY720 Research Evaluating Effects of Daily Oral therapy in Multiple Sclerosis; IFN = interferon; GA = glatiramer acetate; MRI = magnetic resonance imaging; MS = multiple sclerosis; REGAIN = Combination of Antidepressants and Fingolimod RRMS Patients with Depression; RMS = relapsing multiple sclerosis; RRMS = relapsing–remitting multiple sclerosis; SCC = squamous cell carcinoma. STAY = Effect of an Individualized Patient Support Program on Treatment Satisfaction in Fingolimod-treated Patients with RRMS; TOFINGO = Disease Control and Safety in Patients With RRMS Switching From Natalizumab to Fingolimod; TRANSFORMS=TRial Assessing injectable interferoN vS FTY720 Oral in RRMS; UMBRELLA = Long-term Safety and Tolerability of 0.5 mg Fingolimod in Patients With RMS; VZV = varicella zoster virus; x = included in the inclusion or exclusion criteria.

Table S2: Type of centers that patients in PANGAEA were recruited

|  | **Office-based neurologists**  **(%)** | **Multidisciplinary clinics**  **(%)** | **Universities**  **(%)** | **Hospitals**  **(%)** |
| --- | --- | --- | --- | --- |
| Participating centers (n = 265)* | 75.1 | 8.3 | 4.5 | 12.1 |
| Documented patients (n = 3465)^†^ | 66.3 | 7.4 | 13.2 | 13.1 |

*Data were unavailable for 109 centers. ^†^Data were unavailable for 725 patients. PANGAEA = Post-Authorization Non-interventional German sAfety study of GilEnyA.

Table S3 Comorbidities of interest at baseline as a proportion of the number of patients in each cohort

| **Comorbidity** | **PANGAEA baseline** | | |
| --- | --- | --- | --- |
|  | Fingolimod starter cohort *n* = 2532  *n* (%) | Previous study cohort *n* = 730  *n* (%) | Previous study subcohort* *n* = 437 *n* (%) |
| Depression and mood disorders^†^ | 241 (9.5) | 92 (12.6) | 57 (13.0) |
| Hypertension^‡^ | 187 (7.4) | 80 (11.0) | 50 (11.4) |
| Thyroid disease^§^ | 100 (4.0) | 41 (5.6) | 27 (6.2) |
| Migraine and headache^‖^ | 95 (3.8) | 42 (5.8) | 17 (3.9) |
| Diabetes mellitus^¶^ | 50 (2.0) | 5 (0.7) | 3 (0.7) |
| Hypercholesterolemia, hyperlipidemia, hypertrigylceridemia** | 45 (1.8) | 37 (5.1) | 22 (5.0) |
| Increased liver enzyme level^††^ | 44 (1.7) | 9 (1.2) | 6 (1.4) |
| Asthma | 40 (1.6) | 7 (1.0) | 4 (0.9) |
| Epilepsy^‡‡^ | 20 (0.8) | 8 (1.1) | 6 (1.4) |
| Lymphopenia^§§^ | 14 (0.6) | 9 (1.2) | 7 (1.6) |
| Uveitis^‖‖^ | 10 (0.4) | 5 (0.7) | 3 (0.7) |

*The proportion of patients in the previous study cohort who had baseline data available at enrollment into previous fingolimod clinical trials and at enrollment into PANGAEA. ^†^Includes depression, major depression, adjustment disorder, dysthymic disorder, depressed mood, anxiety disorder, mood swings, somatization disorder, generalized anxiety disorder, psychosomatic disease, depressive symptom, personality disorder, psychotic disorder, adjustment disorder with depressed mood, anxiety, mood altered, personality change due to a general medical condition, schizoaffective disorder, and schizophrenia, paranoid type. ^‡^Includes hypertension, blood pressure diastolic increased, blood pressure increased, essential hypertension, and blood pressure measurement. ^§^Includes hypothyroidism, autoimmune thyroiditis, goiter, hyperthyroidism, thyroid disorder, thyroid neoplasm, thyroid function test abnormal, thyroidectomy, and benign neoplasm of the thyroid gland. ^‖^Includes migraine, headache, tension headache, migraine with aura, migraine without aura, and cluster headache. ^¶^Includes diabetes mellitus, type 2 diabetes mellitus, and type 1 diabetes mellitus. ^**^Includes hypercholesterolemia, hyperlipidemia, blood triglycerides increased, low-density lipoprotein increased, blood cholesterol increased, low-density lipoprotein, blood cholesterol, hypertriglyceridemia, blood triglycerides, and lipids increased. ^††^Includes γ-glutamyltransferase increased, hepatic enzyme increased, alanine aminotransferase increased, transaminases increased, aspartate aminotransferase increased, alanine aminotransferase, aspartate aminotransferase, blood bilirubin increased, and γ-glutamyltransferase. ^‡‡^Includes epilepsy, seizure, generalized tonic–clonic seizure, partial seizures, and tonic convulsion. ^§§^Includes lymphopenia, lymphocyte count decreased, and white blood cell count decreased. ^‖‖^Includes uveitis and autoimmune uveitis. PANGAEA = Post-Authorization Non-interventional German sAfety study of GilEnyA.

**Table S4** Concomitant medications at baseline as a proportion of the total number of concomitant medications in each patient cohort

| **Medication class*** | **Number of patients receiving the concomitant medication, n (%)** | | |
| --- | --- | --- | --- |
|  | Fingolimod starter cohort *n* = 2532 | Previous study cohort  *n* = 730 | Previous study subcohort† *n* = 437 |
| Analgesics | 482 (13.4) | 162 (13.2) | 104 (13.5) |
| Antidepressants | 366 (10.2) | 143 (11.7) | 81 (10.5) |
| Corticosteroids | 331 (9.2) | 86 (7.0) | 61 (7.9) |
| Sex steroid hormones | 225 (6.3) | 56 (4.6) | 62 (8.0) |
| Anti-infectives | 171 (4.8) | 89 (7.3) | 47 (6.1) |
| Anticonvulsants | 151 (4.2) | 55 (4.5) | 29 (3.8) |
| Antispastics | 110 (3.1) | 21 (1.7) | 10 (1.3) |
| Bladder medications | 105 (2.9) | 22 (1.8) | 14 (1.8) |
| ACE inhibitors | 96 (2.7) | 31 (2.5) | 21 (2.7) |
| Proton pump inhibitors | 83 (2.3) | 27 (2.2) | 28 (3.6) |
| Fampridine | 82 (2.3) | 11 (0.9) | 4 (0.5) |
| Angiotensin receptor blockers | 59 (1.6) | 29 (2.4) | 15 (1.9) |
| β-blockers | 58 (1.6) | 25 (2.0) | 19 (2.5) |
| Antidiabetics | 50 (1.4) | 4 (0.3) | 1 (0.1) |
| Diuretics | 37 (1.0) | 18 (1.5) | 10 (1.3) |
| Calcium channel blockers | 35 (1.0) | 12 (1.0) | 4 (0.5) |
| Cannabinoids | 22 (0.6) | 8 (0.7) | 2 (0.3) |
| Other | 1135 (31.5) | 426 (34.8) | 259 (33.6) |

*A patient can report more than one concomitant medication. The total number of concomitant medications reported were: fingolimod starter cohort, *n* = 3598; previous study cohort, *n* = 1225; previous study subcohort, *n* = 771. ^†^The proportion of patients in the previous study cohort who had baseline data available at enrollment into previous fingolimod clinical trials and at enrollment into PANGAEA (Post-Authorization Non-interventional German sAfety study of GilEnyA). ACE = angiotensin converting enzyme.

# Supplementary figure legends

Fig. S1 Locations of centers from which patients in PANGAEA were recruited

PANGAEA = Post-Authorization Non-interventional German sAfety study of GilEnyA.

**Fig. S2** Age distribution of patients at entry into PANGAEA

*The proportion of patients in the previous study cohort who had baseline data available at enrollment into previous fingolimod clinical trials and at enrollment into PANGAEA. PANGAEA = Post-Authorization Non-interventional German sAfety study of GilEnyA.

Fig. S3 EDSS score distribution of patients at entry into PANGAEA

*The proportion of patients in the previous study cohort who had baseline data available at enrollment into previous fingolimod clinical trials and at enrollment into PANGAEA. EDSS = Expanded Disability Status Scale; PANGAEA = Post-Authorization Non-interventional German sAfety study of GilEnyA.
