# Supplementary figures and images for: Clinical and Demographic Profile of Patients Receiving Fingolimod in Clinical Practice in Germany and the Benefit–Risk Profile of Fingolimod After 1 Year of Treatment: Initial Results From the Observational, Noninterventional Study PANGAEA
Source: Neurotherapeutics. 2017 Dec 22;15(1):190–9. doi: 10.1007/s13311-017-0595-y (PMC5794706; doi:10.1007/s13311-017-0595-y)

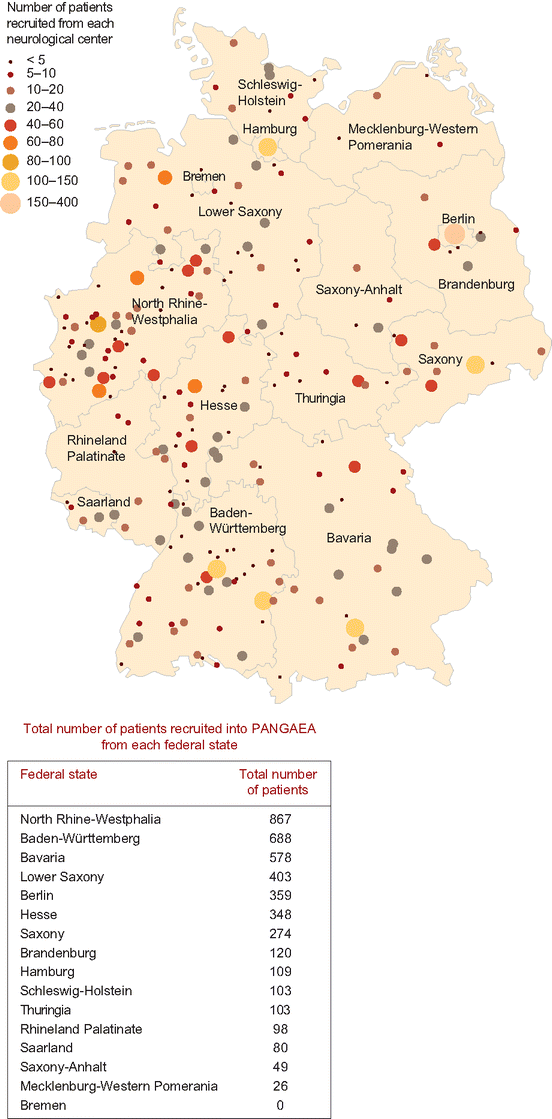

Supplement: Supplementary file 2 — (GIF 118 kb) [file 13311_2017_595_Fig5_ESM.gif]

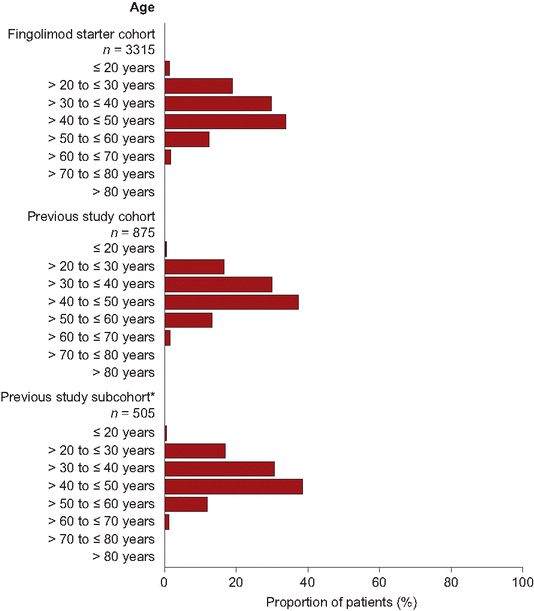

Supplement: Supplementary file 4 — (GIF 36 kb) [file 13311_2017_595_Fig6_ESM.gif]

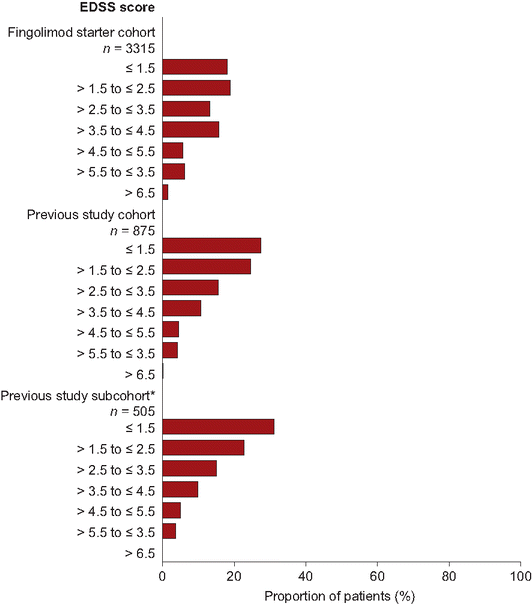

Supplement: Supplementary file 6 — (GIF 26 kb) [file 13311_2017_595_Fig7_ESM.gif]
